# Increased Aβ pathology in aged Tg2576 mice born to mothers fed a high fat diet

**DOI:** 10.1038/srep21981

**Published:** 2016-02-25

**Authors:** Shereen Nizari, Roxana O. Carare, Cheryl A. Hawkes

**Affiliations:** 1 Dept. of Life, Health and Chemical Sciences, Faculty of Science, Open University, Walton Hall, Milton Keynes, UK; 2 Clinical and Experimental Sciences, Faculty of Medicine, University of Southampton, Southampton, UK

## Abstract

Maternal obesity is associated with increased risk of developing diabetes, obesity and premature death in adult offspring. Mid-life diabetes, hypertension and hypercholesterolaemia are risk factors for the development of sporadic Alzheimer’s disease (AD). A key pathogenic feature of AD is the accumulation of β-amyloid (Aβ) in the brain. The purpose of this study was to investigate the effect of high fat diet feeding during early life on Aβ pathology in the Tg2576 mouse model of AD. Female mice were fed a standard (C) or high fat (HF) diet before mating and during gestation and lactation. At weaning, male offspring were fed a C diet. Significantly higher levels of guanidine-soluble Aβ and plaque loads were observed in the hippocampi of 11-month old Tg2576 mice born to mothers fed a HF diet. Changes in the extracellular matrix led to increased retention of Aβ within the parenchyma. These data support a role for maternal and gestational health on the health of the aged brain and pathologies associated with AD and may provide a novel target for both the prevention and treatment of AD.

Over 2.1 billion adults are currently estimated to be overweight or obese, of which approximately 38% are women of childbearing age^1^. Obesity is associated with an intake of diets that are high in saturated fat, typical of a Western diet^2^,^3^. A large body of experimental and epidemiological evidence supports a relationship between maternal diet, gestational environment and health in adulthood^4^. In particular, maternal obesity is associated with increased risk of coronary heart disease, diabetes, obesity, attention deficit hyperactivity disorder and premature death in adult offspring^5^,^6^,^7^. However, there is sparse evidence on how maternal obesity affects the susceptibility to neurodegenerative diseases such as Alzheimer’s disease (AD).

In addition to advanced age, the presence of diabetes, hypertension and hypercholesterolemia in mid-life increases the risk of developing AD^8^. AD is characterized pathologically by the intracellular accumulation of tau and the extracellular deposition of β-amyloid (Aβ) peptides in the parenchyma as plaques and in the walls of arteries and capillaries as cerebral amyloid angiopathy (CAA)^9^. Aβ42 is predominantly found in parenchymal plaques, while CAA is composed principally of Aβ40^10^. Aβ peptides of 38–42 amino acids are produced following the proteolytic cleavage of the amyloid precursor protein (APP) by β- and γ-secretase. Removal of Aβ from the brain is mediated by several mechanisms, including enzymatic cleavage by neprilysin and insulin degrading enzyme (IDE), uptake by microglia and perivascular macrophages, transcytosis across the blood-brain barrier and perivascular drainage along basement membranes of capillaries and arteries^11^,^12^,^13^,^14^,^15^,^16^.

Cerebrovascular basement membranes (CVBM) are specialized sheets of extracellular matrix that are composed of laminins, collagen IV and heparin sulphate proteoglycans such as perlecan and fibronectin and are in direct communication with the extracellular spaces of the parenchyma^17^. The composition of CVBM and the extracellular matrix influence the kinetics of Aβ aggregation. In particular, laminin, nidogens and collagen IV inhibit the aggregation of Aβ and destabilize pre-formed fibrils, while fibronectin and perlecan accelerate the aggregation of Aβ via high-affinity interactions^18^,^19^,^20^. In the aged and AD brain, there is a shift in the relative expression of basement membrane proteins towards those that promote Aβ aggregation, which contributes to a failure of perivascular drainage of Aβ from the brain and the development of CAA and dementia^13^,^21^,^22^,^23^,^24^,^25^,^26^.

The correlation between maternal obesity and the development of metabolic syndrome in adulthood and the increased risk of AD in individuals with metabolic syndrome in mid-life, suggests that a link may exist between maternal obesity and increased risk of AD. In a previous related study we demonstrated that maternal high fat diet resulted in failure of perivascular clearance of Aβ in normal offspring^27^. The aim of the current study was to investigate the effect of prenatal high fat diet exposure on Aβ pathology in the Tg2576 mouse model of AD.

## Results

### Prenatal HF increases levels of guanidine-soluble Aβ40 and Aβ42

**Figure 1:**
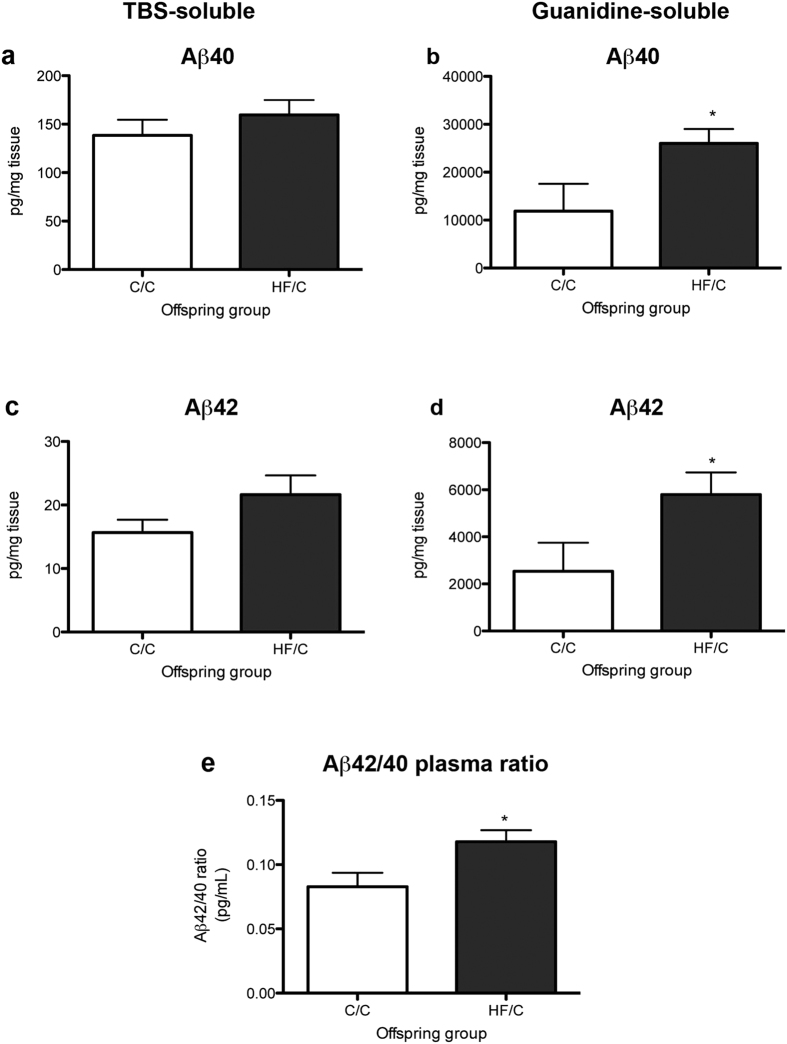
(**a–d**) Levels of hippocampal TBS-soluble human Aβ40 (**a**) and Aβ42 (**c**) did not differ between 11-month old C/C and HF/C Tg2576 mice. However, HF/C mice had significantly higher levels of guanidine-soluble Aβ40 (**b**) and Aβ42 (**d**) compared to C/C animals. **(e)** The plasma ratio of Aβ42:Aβ40 was also higher in HF/C vs. C/C mice. *p < 0.05.

To determine the effect of early life high fat feeding on levels of Aβ, the hippocampi of 11-month old Tg2576 mice born to mothers fed a normal (C/C) and high fat diet (HF/C) were analysed for TBS- and guanidine-soluble human Aβ40 and Aβ42 by ELISA^28^. No differences were detected in the levels of TBS-soluble Aβ40 or Aβ42 between C/C and HF/C offspring (Fig. 1a,c). However, levels of guanidine-soluble Aβ40 and Aβ42 were both significantly higher in the brains of HF/C mice compared to C/C animals (Fig. 1b,d). Plasma Aβ42/40 ratios were also significantly elevated in HF/C vs. C/C mice (Fig. 1e).

### Parenchymal Aβ40 plaque load and size are increased in HF/C mice

**Figure 2:**
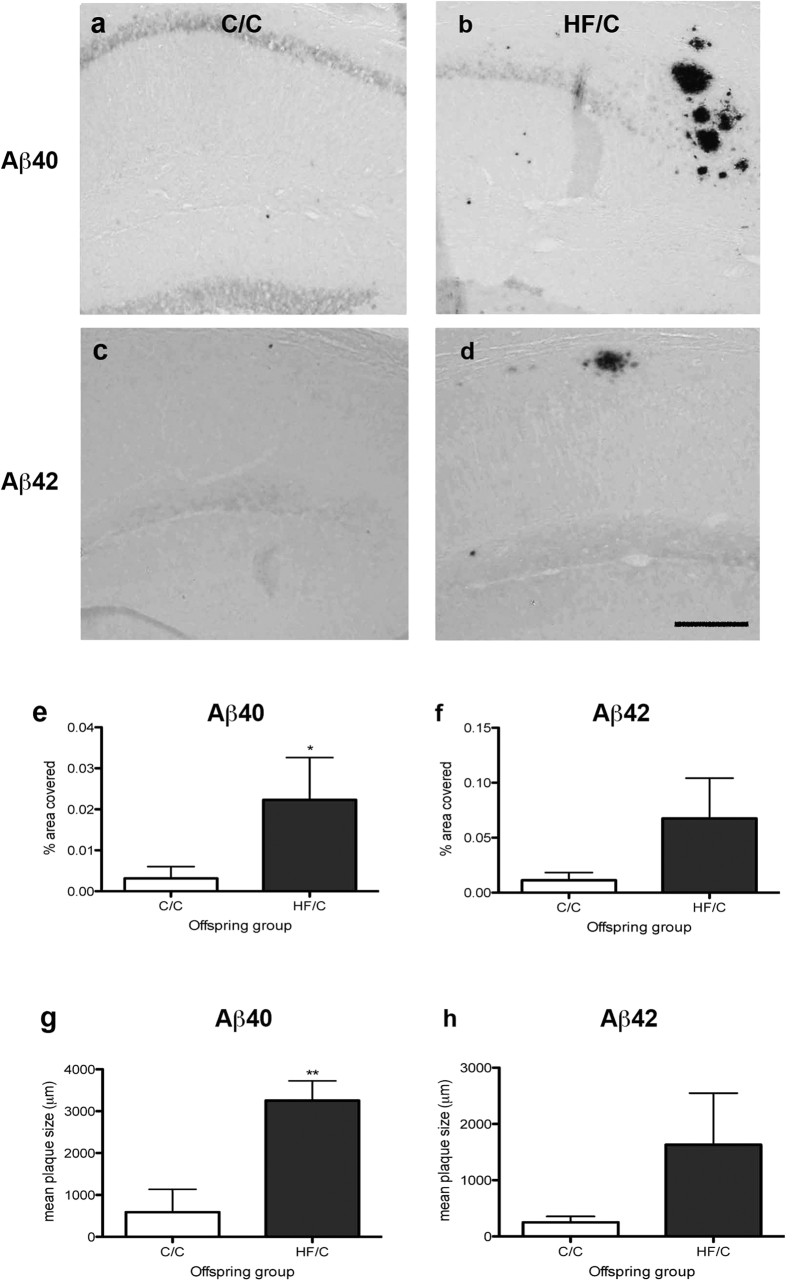
(**a–d**) Photomicrographs of staining of Aβ40- (**a**,**b**) and Aβ42-positive plaques (**c**,**d**) in the hippocampus of C/C (**a**,**c**) and HF/C (**b**,**d**) 11-month old Tg2576 mice. (**e–h**) Both percentage area and average size of Aβ40-positive plaques was significantly higher in HF/C mice compared to C/C offspring (**e, g**) with a similar non-significant trend observed for Aβ42 (**f**,**h**). Scale bar = 500 μm. *p < 0.05.

To confirm that the elevated levels of guanidine-soluble Aβ in HF/C mice were associated with increased Aβ pathology, brains tissue sections were processed for immunohistochemistry using antibodies against human Aβ40 and Aβ42. A few Aβ-positive dense-core parenchymal plaques were observed in the hippocampus of both C/C and HF/C mice, consistent with the amount of insoluble plaque deposition at this age^29^ (Fig. 2a–d). The percentage area covered by Aβ40-positive plaques within the hippocampus was significantly higher in HF/C mice compared to C/C offspring (Fig. 2e). A similar, but non-significant trend was also observed for Aβ42 (Fig. 2f). Analysis of average plaque size showed that Aβ40- and Aβ42-positive plaques were approximately 5.5 and 6.1 times larger, respectively, in HF/C mice than those observed in C/C animals (Fig. 2g,h).

### APP levels are decreased in HF/C mice

**Figure 3:**
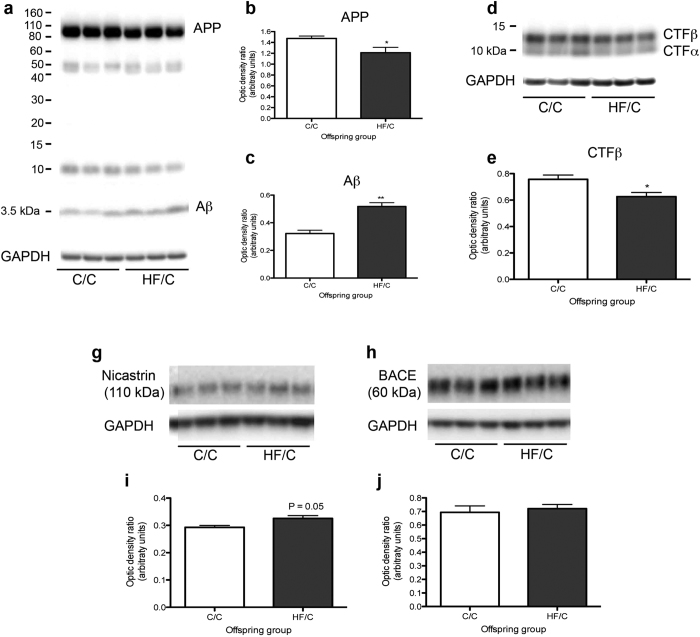
(**a–c**) Levels of full-length APP were significantly decreased and levels of 4 kDa Aβ were increased in the hippocampus of 11-month old Tg2676 HF/C mice compared to C/C mice. (**d**,**e**) Levels of APP-CTFβ were also significantly decreased in the brains of HF/C mice (**d, e**) while APP-CTFα levels were unaltered between diet groups. (**g–j**) An increase in nicastrin expression, which bordered statistical significance (*p* = 0.05), was also noted in HF/C animals (**g–i**). No significant differences were noted in the levels of BACE (**h**,**j**). *p < 0.05, **p < 0.01.

Elevated levels of Aβ may be due to increased APP processing and/or decreased Aβ clearance from the brain. To determine if levels of APP were altered between C/C and HF/C mice, hippocampi from Tg2576 mice were processed by Western blotting with antibodies against full-length and C-terminal fragments of APP (APP-CTF), Aβ1-16, BACE and the γ-secretase component nicastrin. Levels of full-length APP and APP-CTFβ were significantly decreased in the brains of HF/C mice, while levels of 4 kDa Aβ were increased (Fig. 3a–e). An increase in nicastrin expression, which bordered statistical significance (*p* = 0.05), was also noted in HF/C animals (Fig. 3g,i). Levels of BACE did not differ between offspring groups (Fig. 3h,j).

### Diffusion of Aβ within the extracellular matrix is reduced in HF/C mice

**Figure 4:**
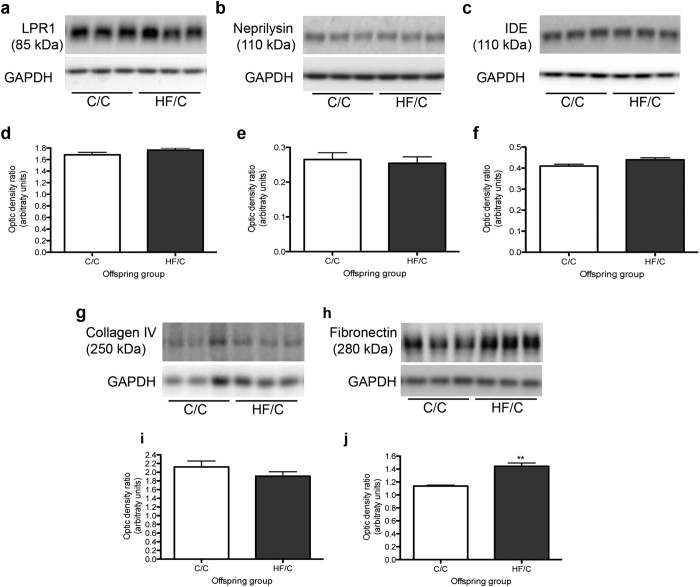
(**a–f**) Levels of hippocampal expression of LRP-1 (**a,d**), neprilysin (**b,e**) and IDE (**c,f**) did not differ between C/C and HF/C mice. **(g–j)** Levels of collagen IV (**g,i**) were also unaltered between offspring groups. However fibronectin levels were significantly higher in the hippocampus of HF/C mice compared to C/C offspring (**h,j**). **p < 0.01.

To determine whether the clearance of Aβ was affected by prenatal exposure to high fat, the levels of enzymes that degrade Aβ (neprilysin, insulin degrading enzyme (IDE)), the endothelial transporter low-density receptor-related protein-1 (LRP-1), as well as basement membrane proteins collagen IV and fibronectin were assessed by Western blotting. No significant differences were noted in the levels of LRP-1, neprilysin or IDE between C/C and HF/C mice (Fig. 4a–f). Collagen IV expression was also unchanged between diet groups (Fig. 4g,i). However, levels of fibronectin were significantly higher in the hippocampi of HF/C mice compared to the C/C group (Fig. 4j,j).

**Figure 5:**
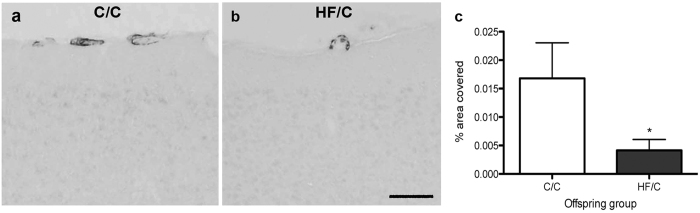
(**a**,**b)** Photomicrographs of Aβ40-positive CAA staining in cortical leptomeningeal vessels of 11-month old C/C (**a**) and HF/C (**b**) Tg2576 mice. **(c)** Vessel coverage by Aβ40-positive staining was significantly lower in HF/C mice than in C/C mice. Scale bar = 500 μm. *p < 0.05.

We have previously shown that cerebrovascular basement membranes are an extension of the parenchymal extracellular matrix^17^ and act as lymphatic drainage pathways along which Aβ is removed from the brain^13^. Failure of this clearance mechanism contributes to the accumulation of Aβ as CAA^25^. To evaluate if vascular Aβ was also affected by prenatal HF exposure, CAA severity was evaluated in cortical leptomeningeal arteries. As demonstrated in Fig. 5, vessel coverage by Aβ40-positive CAA was significantly lower in HF/C mice than in C/C mice (Fig. 5a–c). Quantification of Aβ42-positive vascular deposits was not possible as these deposits were rarely observed in both C/C and HF/C mice.

**Figure 6:**
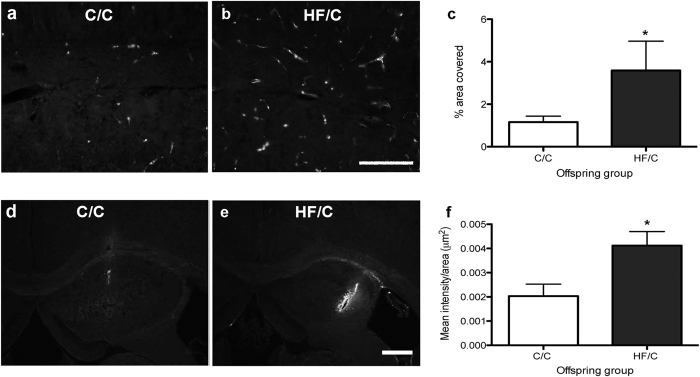
(**a–c**) Photomicrographs of fibronectin staining in the hippocampus of 11-month old C/C (**a**) and HF/C (**b**) Tg2576 mice. Fibronectin covered a significantly greater area in HF/C mice compared to C/C animals (**c**). (**d**,**e**) Photomicrographs of exogenous HilyteFluor-488 human Aβ40 injected into the hippocampus of 5-month old wildtype C/C (**d**) and HF/C mice (**e**). **(f)** Significantly more Aβ was retained at the injection site in the hippocampi of HF/C mice, as indicated by the increased mean fluorescence intensity measured at the site of injection. Scale bars: a and b = 500 μm, d and e = 400 μm. *p < 0.05.

To determine if reduced CAA severity was due to diet-induced changes in the extracellular matrix that increase the retention of Aβ in the parenchyma, we first evaluated the distribution of fibronectin in the hippocampus of C/C and HF/C mice by immunohistochemistry. Consistent with the Western blotting data, fibronectin staining covered a significantly higher percentage of the hippocampus in HF/C mice compared to C/C animals (Fig. 6a–c). To evaluate if the diffusion of Aβ within the extracellular matrix was affected by HF diet independent of plaque pathology, 5-month old wildtype C/C and HF/C mice were injected with human Aβ40. Within 5 minutes post-injection, the majority of the Aβ had diffused away from the injection site of C/C mice (Fig. 6d). Over the same time period, significantly more Aβ was retained in the hippocampi of HF/C mice, as indicated by the increased amount of fluorescence measured at the site of injection (Fig. 6e,f).

## Discussion

Maternal diet is key for the long-term health of the offspring^30^. In the present study, we found that 11-month old Tg2576 mice born to mothers fed a high fat diet during gestation and lactation developed higher levels of guanidine-soluble Aβ and hippocampal plaque load compared to mice born to mothers on a normal diet. Increased Aβ pathology was due in part to a failure of diffusion and increased retention of Aβ within the parenchyma. These data support a key role for early life environment on the health and function of the aged brain and pathologies associated with AD.

The risk of developing sporadic AD is influenced by modifiable factors such as smoking, obesity, and physical and cognitive activity^31^. Among these risk factors, obesity has emerged as a global issue, with approximately 40% of all adults currently estimated to have a body mass index over 25kg/m^2 ^1. The majority of studies investigating the link between obesity and risk of AD have used models of postnatal high fat diet feeding. Pathological features related to AD, in association with decreased cognitive function have been reported in various transgenic mouse models of AD following consumption of diets high in saturated fats and/or cholesterol^32^,^33^,^34^,^35^.

Despite the established link between maternal obesity and the development in adulthood of conditions that themselves increase the risk of AD, such as obesity and diabetes^8^,^36^, very little work has been done to investigate the relationship between maternal obesity and risk of AD. We have previously reported that exposure to a high fat diet during brain development affects the expression of markers of the neurovascular unit and impairs the efficiency of Aβ clearance from the brain along cerebral basement membranes in adult offspring^27^. The present study expands upon these findings by demonstrating that Aβ pathology is higher in Tg2576 mice born to obese mothers, due in part to increased retention of Aβ in the parenchyma. Although Aβ production may also have been affected in HF/C mice, more detailed experiments are needed to definitively determine if the observed increase in levels of 4-kDa Aβ were due to increased APP processing or increased retention.

The findings of this study are in contrast to those of Martin *et al.*^37^, who reported decreased cognitive performance of 12-month old female 3xTg mice exposed to a high fat diet during gestation and lactation in the absence of changes in Aβ plaque load. Although the reasons for this discrepancy are unknown, they may relate to differences in diet composition, sex and/or transgene expression. Nevertheless, both studies support a role for maternal obesity in the etiology of AD-related pathology.

We have previously demonstrated that Aβ contained with the interstitial fluid of the brain is rapidly cleared from the parenchyma along cerebrovascular basement membranes which invaginate the extracellular space^13^,^17^,^38^. Impaired elimination of Aβ and other metabolites along perivascular pathways is hypothesized to trigger the rise in Aβ oligomers that drives the amyloid cascade with seeding of Aβ plaques, hyperphosphorylation of tau and propagation of neurofibrillary tangles, leading to synaptic dysfunction and death^39^. Changes in the glycoprotein and proteoglycan composition of the basement membrane and extracellular matrix influence the kinetics of Aβ aggregation and the efficiency by which Aβ is removed from the brain^21^,^22^,^23^,^24^,^25^. In particular, incubation of Aβ with fibronectin *in vitro* accelerates fibril formation^21^ and increased expression of vascular fibronectin has been shown to precede the development of CAA *in vivo*^40^. In the present study, fibronectin expression was increased in HF/C mice in association with decreased diffusion of Aβ within the extracellular matrix at 5 minutes after injection, suggesting that Aβ was retained within the parenchyma of HF/C mice. This is further supported by the findings that the parenchymal plaques in HF/C mice were significantly larger than those in C/C mice and that CAA severity in cortical leptomeningeal vessels was decreased in HF/C mice. This is in agreement with our previous findings that perivascular drainage of Aβ is disrupted in the presence of pre-existing plaques, which are bound by solutes contained within the ISF^25^. Collectively, these results suggest that early life exposure to a HF diet resulted in alterations in the extracellular matrix and lead to the retention of Aβ within the parenchyma. However, as the present study used only one time point to evaluate the diffusion of Aβ from the parenchyma, further work is needed to determine the time-course of Aβ clearance from the brains of HF/C mice.

Findings from the present study support the Latent Early-Life Associated Regulation hypothesis of AD proposed by Lahiri *et al.*^41^,^42^,^43^, which suggests that maternal diet influences early epigenetic changes that contribute to disease susceptibility in late life. As the incidence of obesity continues to rise in both girls and women of childbearing age^1^, it is imperative that the epigenetic and biochemical pathways that are influenced by maternal obesity are identified. Such information may provide a novel target for both the prevention and treatment of AD.

## Methods

### Mouse prenatal high fat feeding

Female C57Bl/6 and SJLxC57Bl/6 mice were fed a standard (C, 20% kcal fat, 17% kcal protein, 63% kcal carbohydrate, n = 6) or high fat diet (HF, 45% kcal fat, 20% kcal protein, 35% kcal carbohydrate; Special Diet Services, UK, n = 6), for four weeks before mating, as described previously^27^. Females were then mated with either wildtype or Tg2576 male mice. Tg2576 mice express the human Swedish APP mutation and show elevated levels of SDS-insoluble Aβ starting at 6 months of age^29^. Diffuse parenchymal plaques appear around 10 months of age in the cortex and hippocampus and as CAA in cortical leptomeningeal arteries^29^. Dams were kept on the diet during gestation and lactation, with litters standardized to 8 pups to avoid nutritional biases. Male offspring were fed a C diet from weaning, generating 2 offspring groups, C/C and HF/C, representing the pre- and post-weaning diet. Wildtype mice were aged to 5 months while the Tg2576 offspring were sacrificed at 11 months of age. All animal experiments were approved by the local Animal Welfare and Ethical Review Body at the University of Southampton and carried out in accordance with approved guidelines of the Home Office (30/3095).

### Sandwich Aβ ELISA

Tg2576 mice (n = 3/group) were perfused intracardially with 0.01M phosphate buffered saline (PBS), brains rapidly removed and snap frozen. The hippocampus was dissected and sonicated in TBS extraction buffer (140 mM NaCl, 3 mM KCl, 25 mM Tris (pH 7.4), 1% Nonidet P-40, 5 mM EDTA) containing a protease inhibitor cocktail (Sigma Aldrich, Gillingham, UK). Homogenates were centrifuged (20 820 g, 15 mins, 4 °C) and supernatant collected as the soluble fraction. Pellets were sonicated in 5M guanidine HCl in 50 mM Tris (pH 8.0), incubated for 3 hours at room temperature, spun down (20 820 g, 20 mins, 4 °C) and supernatant collected as the guanidine-soluble fraction. Samples were diluted and analyzed using commercially available sandwich ELISA kits (Life Technologies, Paisley, UK) as per manufacturer’s instructions. Plasma samples obtained immediately after sacrifice were also assessed for Aβ levels. Samples were measured in duplicate, with mean values ± S.E.M. reported for treatment groups.

### Immunohistochemistry

Mice (n = 5/group) were perfused intracardially with 0.01M PBS, followed by 4% paraformaldehyde, brains removed and post-fixed overnight. For enzyme-linked immunohistochemistry, brain tissue sections were washed in 0.01M PBS, treated with formic acid (70%, 30 sec) and incubated overnight at 4 °C with antibodies against human anti-Aβ40 (1:250, Merck Millipore, Watford, UK) and anti-Aβ42 (1:250, Merck Millipore). Sections were washed in 0.01M PBS, incubated with biotinylated anti-rabbit (1:400, Vector Labs, Peterborough, UK) and developed using glucose oxidase enhancement with DAB as chromagen. For fluorescent immunohistochemistry, tissue sections were washed in 0.01M PBS, treated with 1 mg/mL pepsin in 0.2N HCl (45 sec, 37 °C) and incubated overnight at 4 °C with anti-fibronectin (1:400, AbD Serotec, Kidlington, UK). Sections were washed in 0.01M PBS, incubated 2 hrs with AlexaFluor 488-conjugated anti-rabbit (1:200; Life Technologies) and coverslipped with mowiol. Images were captured on a Nikon microscope and exported to Photoshop CS. For quantification of percentage area covered by staining, micrographs were converted to binary images, thresholded to eliminate background staining and evaluated by densitometry using Image J software (NIH, Maryland, USA).

### Western blotting

The hippocampi of Tg2576 mice (n = 3/group) were sonicated in Ripa lysis buffer (20 mM Tris-HCl (pH 8.0), 150 mM NaCl, 1 mM EDTA, 0.1% SDS, 1% Igepal, 50 mM NaF, 1 mM NaVO_3_) containing a protease inhibitor cocktail. Proteins (15–45 μg) were separated by gel electrophoresis on 3–8% Tris-acetate or 10–20% Tris-Tricine gels (Life Technologies), transferred to nitrocellulose membranes and incubated with the following antibodies: anti-APP CTF (1:1000, BioLegend, London, UK), anti-Aβ1-16 (clone 6E10, 1:500, BioLegend), anti-nicastrin (1:500, New England Biolabs, Hitchin, UK), anti-β-secretase-1 (BACE, 1:750, New England Biolabs), anti-low-density receptor-related protein-1 (LRP-1, 1:2500, Insight Biotechnology, Wembley, UK), anti-IDE (1:750, Abcam, Cambridge, UK), anti-neprilysin (1:500, Abcam, Cambridge, UK), anti-fibronectin (1:750, AbD Serotec) and anti-collagen IV (1:500, Sigma Aldrich). Membranes were stripped and reprobed with anti-glyceraldehyde-3-PDH (GAPDH) antibody (1:50,000; Sigma Aldrich) to ensure equal protein loading. Immunoblots were repeated twice per antibody and quantified by densitometry using Image J software. Means ± S.E.M. were calculated as an optical density ratio of protein levels normalized to GAPDH levels.

### Intracerebral injections

Wildtype mice (n = 5/group) were injected stereotaxically with HilyteFluor-488 human Aβ40 (100 μM, 0.5 μL, Anaspec, Fremont, USA) into the left hippocampus (coordinates from Bregma: AP = −1.9 mm; ML = 1.5 mm and DV = 1.7 mm). Injection pipettes were left *in situ* for 2 minutes to prevent reflux and mice were sacrificed 5 minutes post-injection. Mice were perfused intracardially with 0.01M PBS, followed by 4% paraformaldehyde, brains sectioned and coverslipped with Mowiol containing citiflor anti-fading reagent. Images were captured on a Nikon microscope and exported to Image J. The size of Aβ bolus was quantified by subtracting the mean grey value intensity of Aβ at the site of injection from the mean grey value intensity of the contralateral, non-injected hippocampus. Intensity values were then divided by the total hippocampal area of each section and expressed as mean intensity/μm^2^.

### Statistical analysis

All mice were coded and measurements were carried out in a blinded fashion. Data obtained from ELISA and immunohistochemistry experiments were analyzed using a one-tailed Student’s t-test, where Aβ pathology was predicted to be greater in the HF/C group. Western blot and injection site data were analyzed using a two-tailed Student’s t-test. All data are presented as mean ± S.E.M. and in all cases significance was set at *p* < 0.05. Analyses were carried using GraphPad Prizm software.

## Additional Information

**How to cite this article**: Nizari, S. *et al.* Increased Aβ pathology in aged Tg2576 mice born to mothers fed a high fat diet. *Sci. Rep.* **6**, 21981; doi: 10.1038/srep21981 (2016).
